# Cholesterol impacts the formation of huntingtin/lipid complexes and subsequent aggregation

**DOI:** 10.1002/pro.4642

**Published:** 2023-05-01

**Authors:** Alyssa R. Stonebraker, Maryssa Beasley, Sophia Massinople, Michelle Wunder, Peng Li, Stephen J. Valentine, Justin Legleiter

**Affiliations:** ^1^ The C. Eugene Bennett Department of Chemistry West Virginia University Morgantown West Virginia USA; ^2^ Rockefeller Neurosciences Institutes West Virginia University Morgantown West Virginia USA; ^3^ Department of Neuroscience West Virginia University Morgantown West Virginia USA

**Keywords:** amyloid, atomic force microscopy, fibrils, Huntington's disease, lipid binding, mass spectrometry, polyglutamine

## Abstract

Huntington's disease (HD) is a neurodegenerative disease resulting from an expansion of the polyglutamine (polyQ) domain within the huntingtin protein (htt). PolyQ expansion triggers toxic aggregation and alters htt/lipid interactions. The first 17 amino acids at the N‐terminus of htt (Nt17) have a propensity to form an amphipathic α‐helix crucial to aggregation and membrane binding. Htt interacts closely with a variety of membrane systems including those of the endoplasmic reticulum, mitochondria, nuclear envelope, and plasma membrane. Membrane composition heavily influences both htt aggregation and lipid interactions, and cholesterol is a crucial membrane component that modulates properties such as fluidity, permeability, and organization. In HD, cholesterol homeostasis is disrupted, and likely plays a role in toxicity. The objective of these studies was to identify the impact of cholesterol on htt aggregation and lipid interactions in various lipid systems. Lipid systems of POPC, DOPC, and POPG with varied levels of exogenously added cholesterol were exposed to htt, and the influences on aggregation, lipid binding, and htt/lipid complexation were evaluated using thioflavin‐T aggregation assays, atomic force microscopy, colorimetric lipid binding assays, and mass spectrometry. The addition of cholesterol to DOPC vesicles enhanced htt aggregation. In the presence of vesicles of either POPC or POPG, the addition of cholesterol reduced htt aggregation. Htt/lipid binding decreased for POPC and increased for both DOPC and POPG with increasing cholesterol content, with observed differences in htt/lipid complexation. Altered cholesterol content influences htt aggregation, lipid binding, and complexation differently depending on overall lipid composition.

## 1 INTRODUCTION

Huntington's disease (HD) is a fatal neurodegenerative disease caused by an expanded polyglutamine (polyQ) domain in the huntingtin protein (htt; Macdonald, 1993). PolyQ domain expansion beyond a threshold of ~35 repeats (Penney et al., 1997; Snell et al., 1993) results in the aggregation of htt into amyloid‐like fibrils (Chen et al., 2002; Scherzinger et al., 1997, 1999) through a complex pathway that involves additional aggregate species including oligomers and amorphous aggregates (Adegbuyiro et al., 2017). Peptide sequences directly adjacent to the polyQ domain heavily influence the aggregation process (Arndt, Chaibva, & Legleiter, 2015; Burke, Kauffman, et al., 2013; Nagarajan et al., 2014). One such sequence flanking the polyQ domain of htt is the first 17 amino acids at the N‐terminus (Nt17; Arndt, Chaibva, & Legleiter, 2015; Burke, Kauffman, et al., 2013; Chaibva et al., 2016; Kotler et al., 2019; Mishra et al., 2012; Nagarajan et al., 2014). Nt17 accelerates aggregation as it can form an amphipathic α‐helix that promotes early interactions of htt through intermolecular self‐association to form oligomers (Arndt, Chaibva, & Legleiter, 2015; Arndt, Kondalaji, et al., 2015; Kotler et al., 2019). The interprotein association of Nt17 brings polyQ domains into close proximity, promoting fibril nucleation (Kotler et al., 2019; Williamson et al., 2010). The association of Nt17 into complexes on the order of dimers and tetramers was demonstrated by ion mobility spectrometry‐linear ion trap mass spectrometry (IMS‐MS; Arndt, Kondalaji, et al., 2015) and α‐helix rich structures of both Nt17 dimers and tetramers have been resolved by NMR (Kotler et al., 2019).

While a variety of functions have been attributed to htt, many of these, for example, vesicle transport and synaptic transmission (Cattaneo et al., 2005; Li & Li, 2004), require direct interaction with lipid membranes. Htt associates closely with a variety of membranes of varying lipid composition, localizing to the ER (Atwal et al., 2007; De Rooij, 1996; Xia, 2003), mitochondrial (Chang et al., 2006; Choo, 2004; Gu et al., 1996; Orr et al., 2008; Panov et al., 2002), nuclear (Atwal et al., 2007; Xia, 2003), and plasma membranes (Kegel et al., 2005). In addition to promoting aggregation, the propensity for Nt17 to form an α‐helix causes it to facilitate lipid binding (Arndt, Chaibva, & Legleiter, 2015; Burke, Hensal, et al., 2013). As htt readily associates with lipids, the presence of lipid membranes can modify htt aggregation in a lipid composition‐dependent manner (Beasley et al., 2019, 2021, 2022; Chaibva et al., 2018; Côté et al., 2015; Levy et al., 2019; Pandey et al., 2018). Membranes composed of total brain lipid extract (TBLE) inhibit htt fibrilization compared with the absence of lipids (Beasley et al., 2019). Similarly, cellular lipid extracts also reduce htt aggregation (Levy et al., 2019). Enriching TBLE with either sphingomyelin (SM) or ganglioside (GM1) content further modifies htt/lipid interactions and subsequent aggregation, with both SM and GM1 reducing htt insertion into the membrane and promoting distinct aggregate morphologies (Chaibva et al., 2018). However, some pure lipid systems, like palmitoyl‐2‐oleoyl‐glycero‐3‐phosphocholine (POPC; Beasley et al., 2019) or a mixture also containing 1‐palmitoyl‐2‐oleoyl‐sn‐glycero‐3‐phospho‐l‐serine (POPS; Pandey et al., 2018) enhance fibrillization. In particular, the POPS/POPC lipid system promotes a distinct aggregation pathway facilitated by Nt17 compared to aggregation in the absence of lipids (Pandey et al., 2018). Generally, Nt17 displays higher affinity for anionic phospholipids, such as PS and phosphatidylglycerol (PG) lipids, compared to zwitterionic PC lipids (Côté et al., 2015). Beyond head group charge, studies on the impact of unsaturation in lipid tails revealed no direct correlation between htt aggregation and htt/lipid complexation, but that the orientation of Nt17 on the membrane surface is related to membrane defect sizes and how well the hydrophobic residues of Nt17 match those defect sizes (Beasley et al., 2022). Htt has also been shown to cause membrane damage, which is mediated by factors including polyQ length (Burke, Hensal, et al., 2013; Kegel et al., 2005; Riguet et al., 2021), cholesterol content (Gao et al., 2016), and the presence of SM and GM1 (Chaibva et al., 2018). Thus, the distinct physiochemical properties of lipid systems resulting from varied compositions potentially underlie the distinct interactions between htt and different lipid systems.

An important membrane component in the central nervous system is cholesterol, with ~25% of total body cholesterol being found in the brain (Gao et al., 2016; Korade & Kenworthy, 2008; Valenza & Cattaneo, 2011; Vance, 2012). Brain cholesterol is synthesized locally due to the blood–brain barrier preventing uptake from circulation (Korade & Kenworthy, 2008; Valenza, 2005; Valenza & Cattaneo, 2011; Vance, 2012), and cholesterol is critical to effective signal transduction through its roles in myelination (Valenza & Cattaneo, 2011; Vance, 2012) and lipid raft function (Arora et al., 2004; Korade & Kenworthy, 2008; Valenza & Cattaneo, 2011; Vance, 2012). More broadly, cholesterol modulates physical membrane properties, including fluidity and permeability (Subczynski et al., 2017; Wennberg et al., 2012), organization (Arora et al., 2004), and ultimately membrane function (Arora et al., 2004; Subczynski et al., 2017). In HD, cholesterol homeostasis is disrupted, though there are conflicting reports on whether cholesterol content is reduced (Korade & Kenworthy, 2008; Valenza, 2005; Valenza & Cattaneo, 2011; Vance, 2012) or increased (Trushina et al., 2006). When htt was exposed to TBLE membranes enriched in cholesterol, the binding and insertion of htt into membranes were reduced, and membrane susceptibility to htt‐induced damage was decreased (Gao et al., 2016). Interestingly, cholesterol enrichment also promoted a distinct htt aggregation pattern on the membrane surface that resulted in elevated, plateau‐like domains that extended several nanometers above the membrane surface.

Aggregation is heavily influenced by the cellular environment, and htt–lipid interactions are dependent on lipid system and composition. As cholesterol homeostasis is altered in HD, the influence of cholesterol content on htt aggregation and htt/lipid interactions may play a key role in pathogenesis. Our aim was to identify how the addition of cholesterol modulates the ability of htt to bind and complex with different lipid species with varying head groups and tails and evaluate the effect of these interactions on subsequent aggregation.

## 2 RESULTS

### 2.1 Lipids alter htt‐exon1(46Q) aggregation in a composition‐dependent manner

**FIGURE 1:**
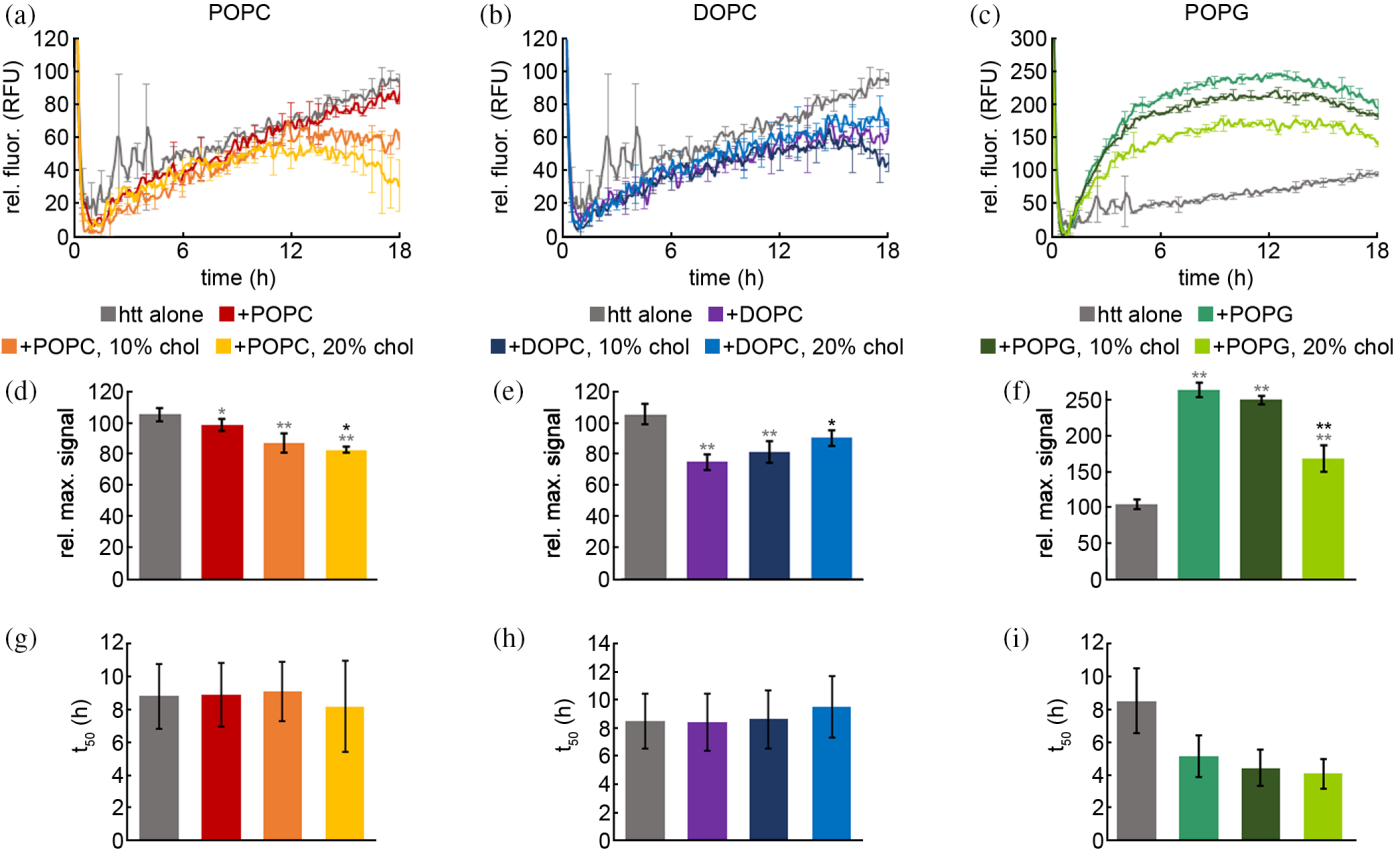
ThT aggregation assays for htt‐exon1(46Q) incubated in the presence of different lipid vesicles with varying amounts of exogenously added cholesterol. Htt concentration was 10 μM with a protein:lipid ratio of 1:10. Representative ThT assays for POPC (a), DOPC (b), and POPG (c) conditions are shown. The relative maximum signal for all POPC (d), DOPC (e), and POPG (f) conditions were calculated and averaged across all runs. The time to reach 50% of the maximum signal (t_50_) for all POPC (g), DOPC (h), and POPG (i) conditions were determined and averaged across all runs. In panels a–c, error bars are provided for every sixth data point (30 min) and represent the standard error of the mean (SEM) between triplicate wells of an individual run. Analysis in panels d–f were determined as averages over all trials, values are normalized as a percentage relative to the htt–exon1(46Q) control in the absence of lipid, and error bars represent SEM. Analysis in panels g and h were determined as averages across all runs and error bars represent SEM. Using a Student's *t*‐test, * represents a *p*‐value <0.05 and ** represents a *p*‐value of <0.01. Gray asterisks are for conditions relative to the htt control, black asterisks are for conditions relative to the pure lipid system.

The ability of lipid membranes to alter aggregation is well established (Beasley et al., 2019, 2021, 2022; Chaibva et al., 2018; Côté et al., 2015; Gao et al., 2016; Levy et al., 2019; Pandey et al., 2018). To determine how the addition of cholesterol impacts the ability of lipid membranes to influence htt aggregation, htt‐exon1(46Q) (10 μM) was incubated with vesicles of POPC, DOPC, or POPG that contained no cholesterol or were enriched with either 10% or 20% cholesterol (% w/w) in a 10:1 lipid to protein molar ratio. The cholesterol content was chosen as it is within the regime most relevant for cellular membranes. Aggregation was monitored as a function of time using ThT, which fluoresces when bound to fibril‐associated β‐sheet structure (Figure 1a–c). Htt‐exon1(46Q) aggregation in the absence of lipids was measured for comparison. The maximum ThT signal for each condition was analyzed, then normalized to htt‐exon1(46Q) alone to compare the extent of fibril formation (Figure 1d–f). The time required to reach 50% of the maximum signal (t_50_) was also determined to evaluate if different lipid systems were altering initial aggregation events (Figure 1g–i).

The effect of lipid vesicles on htt‐exon1(46Q) aggregation was dependent on both lipid composition and the amount of exogenously added cholesterol (Figure 1). Pure POPC vesicles had minimal, though statistically significant, impact on htt‐exon1(46Q) aggregation with a 6% reduction in signal. The subsequent addition of exogenous cholesterol to POPC vesicles had an inhibitory effect, as the addition of 10% and 20% exogenous cholesterol reduced the relative maximum signal by 11% and 16%, respectively, relative to the pure POPC vesicles (Figure 1d); however, there was no statistically significant difference in the t_50_ for all POPC conditions compared to control. The presence of pure DOPC vesicles inhibited htt‐exon1(46Q) aggregation with a 30% reduction in relative maximum signal. Subsequent additions of exogenous cholesterol promoted aggregation relative to the pure lipid system, with 5% and 15% increases in signal for 10% and 20% exogenous cholesterol respectively. Even with some signal recovery for the conditions with exogenous cholesterol, aggregation overall was still reduced by 15%–23% relative to htt‐exon1(46Q) alone (Figure 1e). The t_50_ was unaffected by the presence of DOPC vesicles (with and without cholesterol). Pure POPG vesicles promoted aggregation, with a 160% increase in signal. The addition of exogenous cholesterol reduced aggregation 14%–97% relative to the pure lipid system. Conditions containing exogenous cholesterol still promoted aggregation relative to htt‐exon1(46Q) alone, with a 63%–146% increase in signal (Figure 1f). With POPG vesicles (with and without cholesterol) strongly increasing aggregation, the t_50_ appeared reduced relative to control, but this did not reach statistical significance.

**FIGURE 2:**
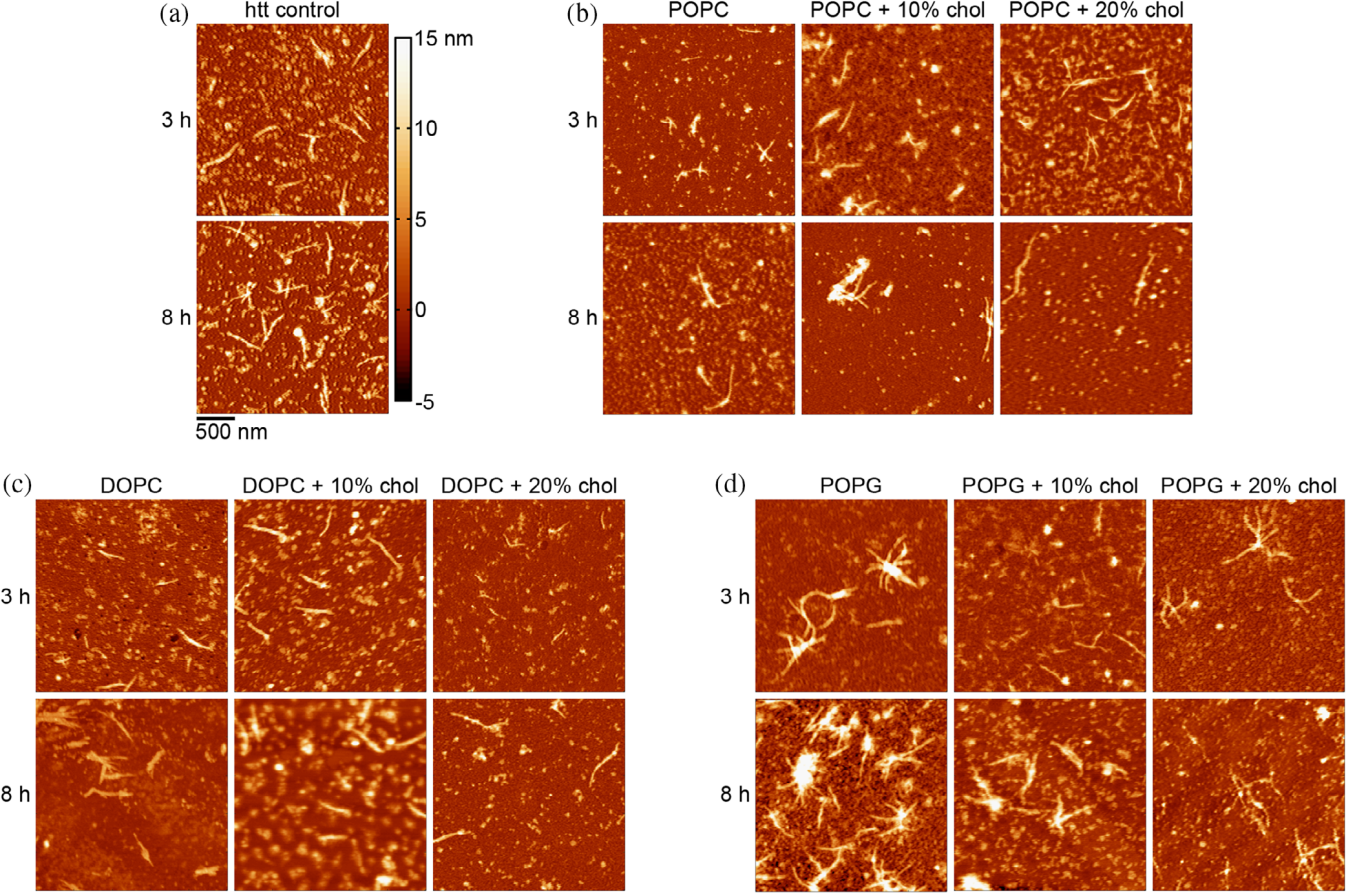
Representative AFM images of 10 μM htt‐exon1(46Q) incubated with no lipid (a), pure POPC and POPC with 10% or 20% exogenous cholesterol (b), pure DOPC and DOPC with 10% or 20% exogenous cholesterol (c), and pure POPG and POPG with 10% or 20% exogenous cholesterol (d). The protein:lipid ratio was 1:10. The colormap and scale bar is applicable to all images.

**FIGURE 3:**
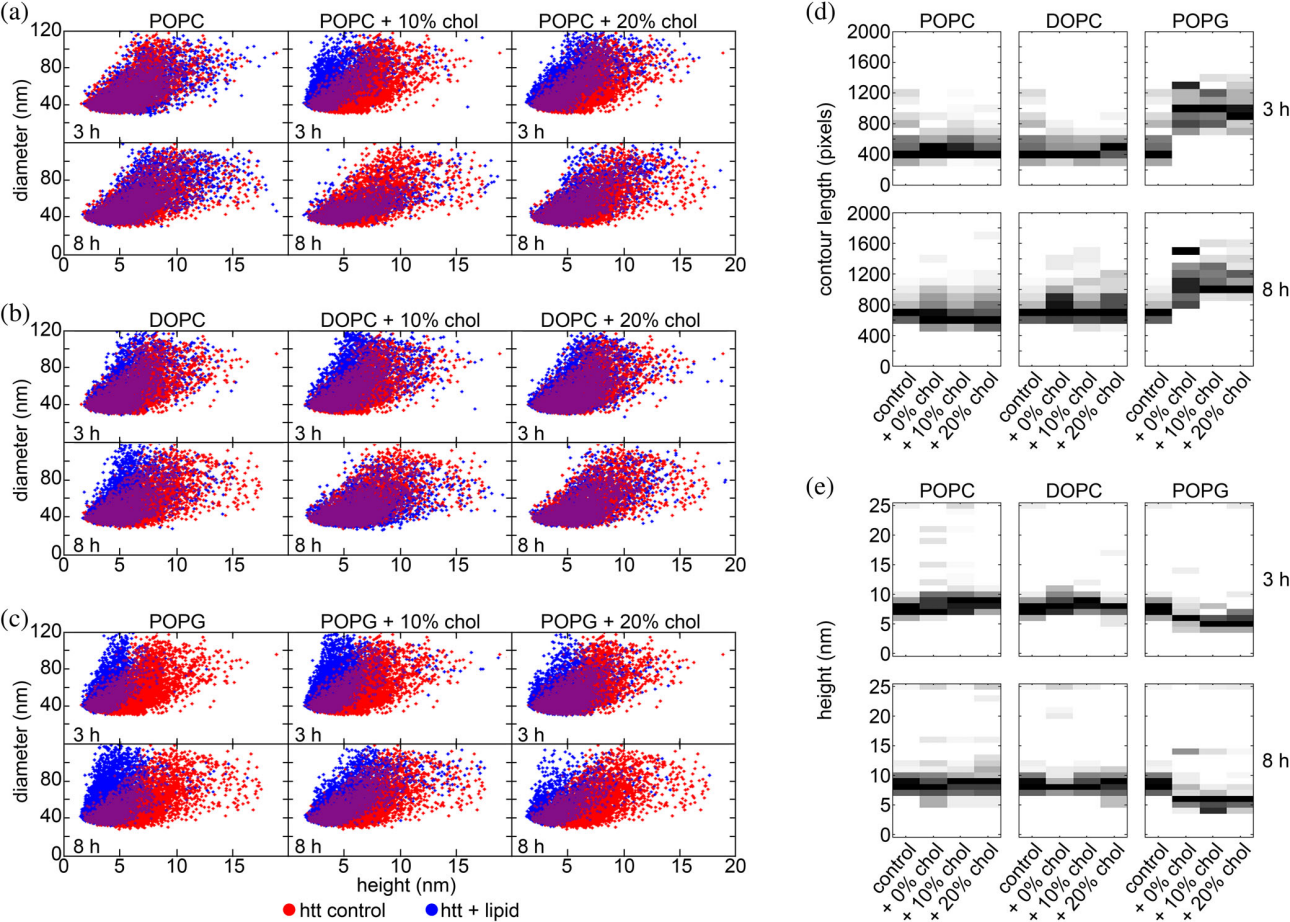
AFM analysis of observed htt‐exon1(46Q) aggregate species under varied lipid conditions. Correlation plots of diameter and height each oligomer at 3 h for htt‐exon1(46Q) in the absence of lipid and incubated in the presence of pure POPC and POPC with 10% and 20% exogenous cholesterol (a), pure DOPC and DOPC with 10% and 20% exogenous cholesterol (b), pure POPG and POPG with 10% and 20% exogenous cholesterol (c). Histograms of fibril heights comparing htt‐exon1(46Q) in the absence of lipid to all POPC lipid conditions (d), all DOPC lipid conditions (e), and all POPG lipid conditions (f).

To further investigate the effect of lipid composition and exogenous cholesterol content on htt‐exon1(46Q) aggregation, morphology of htt aggregates was evaluated using AFM (Figures 2 and 3). Freshly prepared htt‐exon1(46Q) (10 μM) was incubated in the presence and absence of vesicles composed of POPC, DOPC, or POPG (10:1 lipid:htt‐exon1(46Q) molar ratio). Additional incubations were performed with vesicles of each lipid that contained either 10% or 20% exogenously added cholesterol. The 3 h time point was chosen because it typically results in a large oligomer population. The 8 h time point was chosen to obtain images of fibrils before extensive bundling, branching, and crossing occurred. Both oligomers and fibrils were observed across all incubation conditions (Figure 2). Fibril morphology appeared consistent with the htt‐exon1(46Q) control for all POPC and DOPC lipid systems (Figure 2a–c), while a distinct spider‐like fibril morphology was observed in the presence of all POPG lipid conditions (Figure 2d). Considering this observation, oligomer and fibril morphologies under each lipid condition were evaluated using automated Matlab scripts that determined morphological features for each individual aggregate. An oligomer was defined as any feature within an image composed of fewer than 50 pixels. For fibril analysis, fibrils were defined as any feature larger than 50 pixels (large amorphous aggregates were excluded by hand) with a contour length of at least 200 nm. In the case of the unique spider‐like morphology observed under all POPG lipid conditions, the centers of the aggregates were much taller than the protruding fibrils, and thus were excluded for analysis of fibril height. The branching of POPG fibrils associated with the star‐like morphology complicated the analysis of contour length. As a result, contour length represents the longest path through each star‐like fibril.

To deeper analyze if the lipid systems altered oligomer formation, oligomer morphology was evaluated by comparing the diameters and heights of oligomers identified in the htt‐exon1(46Q) control at both the 3 and 8 h time points to those identified when incubated with each lipid system (Figure 3a–c). Oligomers formed in the presence of pure POPC were not morphologically different from those formed in the absence of lipid at either 3 or 8 h; however, the addition of 10% or 20% cholesterol to POPC vesicles promoted a statistically significant shift to smaller oligomer heights at 3 h (*p* < 0.01 for each condition, Chi‐squared test) while 8 h oligomers were not significantly different from the control (Figure 3a, Table 1). Under all DOPC lipid conditions, at both 3 and 8 h, oligomers were not morphologically different from the htt‐exon1(46Q) control (Figure 3b, Table 1). Under all POPG lipid conditions, at both 3 and 8 h, oligomers shifted to significantly smaller heights (*p* < 0.01 for all conditions; Figure 3c, Table 1).

**TABLE 1:** Significance of oligomer height, fibril height, and fibril contour length relative to the htt control.

Condition	3 h Significance^a^	8 h Significance^a^
Oligomer height		
htt control	N/A	N/A
POPC 0% cholesterol	Not significant	Not significant
POPC 10% cholesterol	significant	Not significant
POPC 20% cholesterol	significant	Not significant
DOPC 0% cholesterol	Not significant	Not significant
DOPC 10% cholesterol	Not significant	Not significant
DOPC 20% cholesterol	Not significant	Not significant
POPG 0% cholesterol	Significant	Significant
POPG 10% cholesterol	Significant	Significant
POPG 20% cholesterol	Significant	Significant
Fibril contour length		
htt control	N/A	N/A
POPC 0% cholesterol	Not significant	Not significant
POPC 10% cholesterol	Not significant	Not significant
POPC 20% cholesterol	Not significant	Not significant
DOPC 0% cholesterol	Not significant	Not significant
DOPC 10% cholesterol	Not significant	Not significant
DOPC 20% cholesterol	Not significant	Not significant
POPG 0% cholesterol	Significant	Significant
POPG 10% cholesterol	Significant	Significant
POPG 20% cholesterol	Significant	Significant
Fibril height		
htt control	N/A	N/A
POPC 0% cholesterol	Not significant	Not significant
POPC 10% cholesterol	Not significant	Not significant
POPC 20% cholesterol	Not significant	Not significant
DOPC 0% cholesterol	Not significant	Not significant
DOPC 10% cholesterol	Not significant	Not significant
DOPC 20% cholesterol	Not significant	Not significant
POPG 0% cholesterol	Significant	Significant
POPG 10% cholesterol	Significant	Significant
POPG 20% cholesterol	Significant	Significant

^a^ A condition is considered significant if the result from a Chi‐squared test was *p* < 0.01.

Fibril morphologies were compared by measuring the contour lengths of fibrils and the average height along the contour of the fibril. As the POPG star‐like fibril morphology had a large height at the center where the branches connected, this was removed in calculating the average height along the contour. For branching structures, the longest path through the aggregate was considered the contour length. Fibril contour lengths for all POPC and DOPC systems were not significantly different from the htt‐exon1(46Q) control at either 3 or 8 h, while the POPG system shifted toward significantly longer fibrils at both timepoints (Figure 3d, Table 1). The control demonstrated mode fibril contour lengths of 400 nm at 3 h, which was consistent for all POPC and DOPC systems regardless of cholesterol content while POPG fibrils shifted to longer contour lengths of mode 1000 nm. Fibril contour length increased at 8 h for all conditions, with the control shifting to a mode length of 700 nm and the POPC and DOPC conditions, regardless of cholesterol content, demonstrating minimal deviations from the control. The POPG system contour lengths remained relatively consistent under all conditions with a mode of 1000–1100 nm. Much like with contour length, fibril heights along the contour for all POPC and DOPC systems were not significantly different from the htt‐exon1(46Q) control at either 3 or 8 h, while the POPG systems shifted toward significantly smaller heights (*p* < 0.01) at both timepoints (Figure 3e, Table 1). Overall heights were consistent at both the 3 and 8 h timepoints, with POPC and DOPC conditions, regardless of cholesterol content, demonstrating a mode height of approximately 7–8 nm, while POPG shifted smaller to mode heights of 5–6 nm. Generally, aggregate morphologies for the zwitterionic lipid systems were more consistent across all conditions relative to the htt‐exon1(46Q) control. The most notable change was with the anionic POPG systems, where oligomers were typically smaller and fibrils displayed a unique morphology with increased contour length and reduced fibril height.

### 2.2 Htt‐exon1(46Q)/lipid interactions are altered in a composition‐dependent manner

**FIGURE 4:**
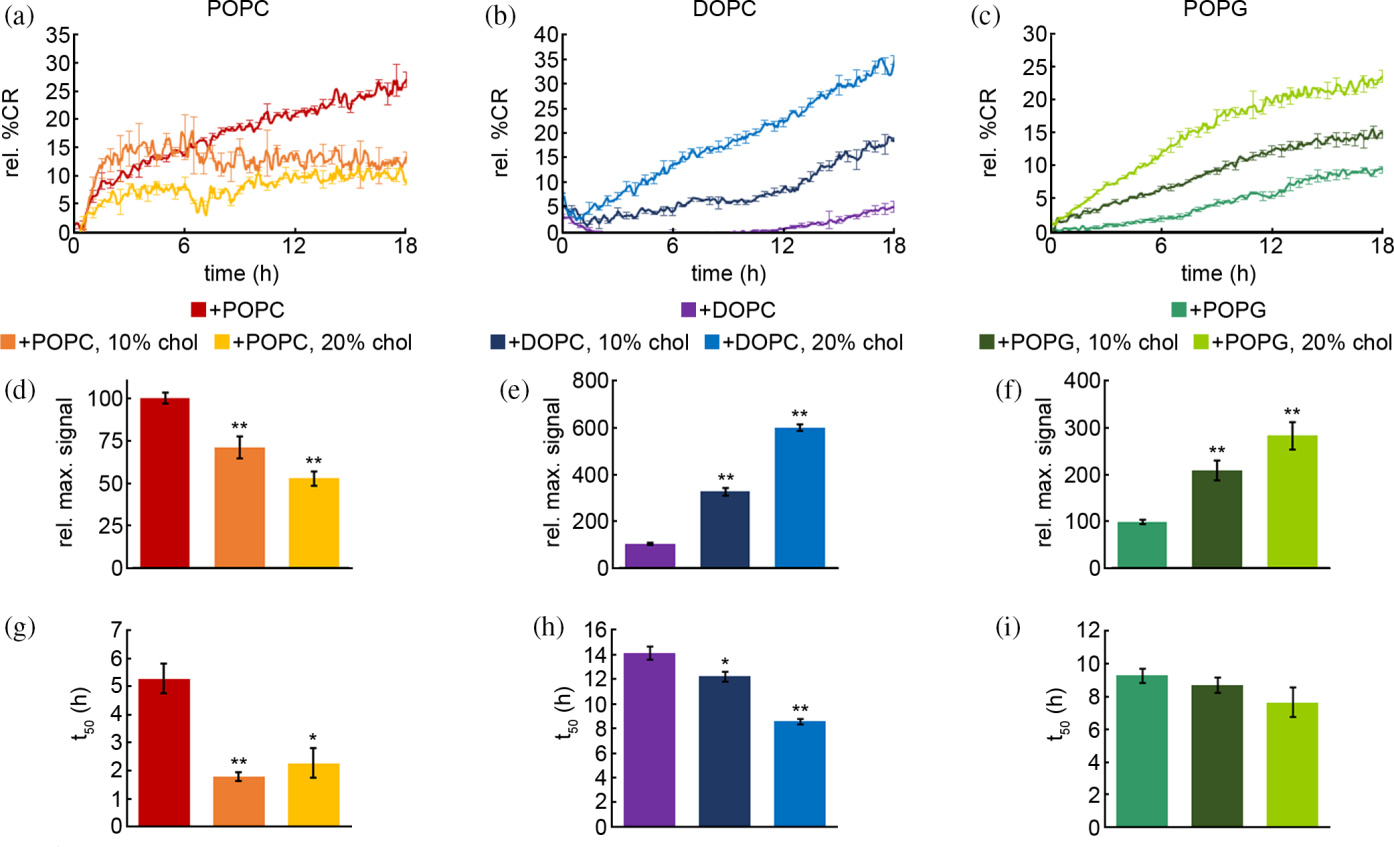
PDA/lipid binding assays for htt‐exon1(46Q) incubated with different lipid systems with varied amounts of exogenously added cholesterol. Htt concentration was 10 μM with a protein:lipid ratio of 1:10. Representative PDA assays for POPC (a), DOPC (b), and POPG (c) conditions are shown. The relative maximum signal for all POPC (d), DOPC (e), and POPG (f) conditions were calculated and averaged across all runs. The time to reach 50% of the maximum signal (t_50_) for all POPC (g), DOPC (h), and POPG (i) conditions were determined and averaged across all runs. In panels a–c, error bars are provided for every sixth data point (30 min) and SEM between triplicate wells of an individual run. Analysis in panels d–f were determined as averages over all trials, values are normalized as a percentage relative to the htt‐exon1(46Q) control in the presence of pure lipid, and error bars represent SEM. Analysis in panels g and h were determined as averages across all runs and error bars represent SEM. Using a Student's *t*‐test, * represents a *p*‐value of <0.05 and ** represents a *p*‐value of <0.01 relative to the pure lipid systems.

Cholesterol content was shown to impact aggregation to different extents depending on lipid composition, indicating altered interactions between htt‐exon1(46Q) and lipids. To directly evaluate htt‐exon1(46Q) interactions with lipids and the influence of exogenous cholesterol, a colorimetric membrane binding assay with lipid/polydiacetylene (PDA) vesicles was used. The lipid components were POPC, DOPC, POPG, or the same pure lipid systems with 10% or 20% exogenously added cholesterol. When exposed to proteins, the colorimetric response (CR) of each system directly correlates to the extent of protein/lipid interaction, as there is a transition in the structure of the polymerized PDA backbone due to the binding and/or insertion of proteins into the vesicle (Figure 4a–c). The percent CR was obtained for each condition as described by Equation 1. The maximum percent CR was then calculated by dividing the percent CR of the designated condition by the maximum percent CR from exposure of the lipid vesicles to NaOH, which was then normalized to 100%. Relative percent CR of each well was calculated by dividing the maximum percent CR of each well by the average maximum percent CR across triplicate wells of that condition normalized to 100%, which were then averaged across all separate runs (Figure 4d–f). The time required to reach 50% of the maximum signal (t_50_) was also determined to evaluate the relative rate of htt‐exon1 insertion into the vesicles (Figure 4g–i).

Incubation of htt‐exon1(46Q) with POPC vesicles containing 10% and 20% cholesterol resulted in a decrease in the relative maximum signal by 30% and 48% respectively compared to interactions with pure POPC vesicles, indicating decreasing htt‐exon1(46Q)/lipid interactions with increasing cholesterol content (Figure 4d). Despite the total binding signal reducing with the addition of cholesterol to POPC vesicles, the t_50_ was significantly reduced relative to the pure POPC system. This suggests that cholesterol alters the membrane in a manner that promotes accessible binding sites, but that these sites are quickly saturated (Figure 4g). When htt‐exon1(46Q) was incubated with DOPC vesicles containing 10% and 20% cholesterol there was an increase in the relative maximum signal of 225% and 500%, respectively, relative to interactions with pure DOPC vesicles, indicating increased htt‐exon1(46Q)/lipid interactions with increasing cholesterol content (Figure 4e). The t_50_ for DOPC significantly decreased with the addition of cholesterol, suggesting that the addition of cholesterol creates more putative binding sites on the vesicle surface but that these sites are not necessarily more accessible (Figure 4h). In the case of POPG vesicles with 10% and 20% cholesterol, an increase in relative maximum signal by 110% and 184% respectively relative to interactions with pure POPG vesicles, indicating increased htt‐exon1(46Q)/lipid interactions with increasing cholesterol content (Figure 4f). While the t_50_ decreased with the addition of cholesterol into POPG vesicles (Figure 4i), this change was not statistically significant.

### 2.3 Cholesterol content alters complex formation between lipids and the lipid binding domain of htt‐exon1(46Q)

The Nt17 domain of htt‐exon1(46Q) facilitates interactions with lipid and can modify aggregation on membranes (Burke, Kauffman, et al., 2013). Nt17 peptide has been shown to have a similar interaction mechanism with membrane as htt‐exon1(46Q) (Pandey et al., 2018), and the mechanism does not change significantly with the addition of glutamines (Côté et al., 2014). Therefore, synthetic Nt17 peptide was used to investigate the influence of lipid composition on the formation of protein/lipid complexes using capillary vibrating sharp‐edge spray ionization (cVSSI)‐MS due to the demonstrated ability of cVSSI to increase ion signals for field‐enabled experiments in both positive and negative mode (Li et al., 2020). Nt17 (10 μM) was incubated with vesicles of POPC, DOPC, POPG, and the same pure lipid systems with 20% cholesterol, at a 10:1 lipid‐to‐peptide molar ratio for 24 h prior to analysis with cVSSI‐MS. The 24 h incubation time was chosen based on work using another small peptide, melittin, in which acyl chain transfer from lipid to peptide was observed in the presence of cholesterol (Britt et al., 2019), allowing for the observation of potential cholesterol‐aided acyl chain transfer to Nt17. Triplicate spectra of each sample were collected in positive ion mode. The integrated peak area of each identified complex was calculated by integrating the ion signal for each isotopic distribution of the given complex. The integrated peak areas of all complexes were normalized to that of the doubly‐charged peptide monomer ([M + 2H]^2+^ at *m*/*z* 988.50). Complexes are represented in [M + L] format, in which M represents Nt17 peptide and L represents the specified lipid.

**FIGURE 5:**
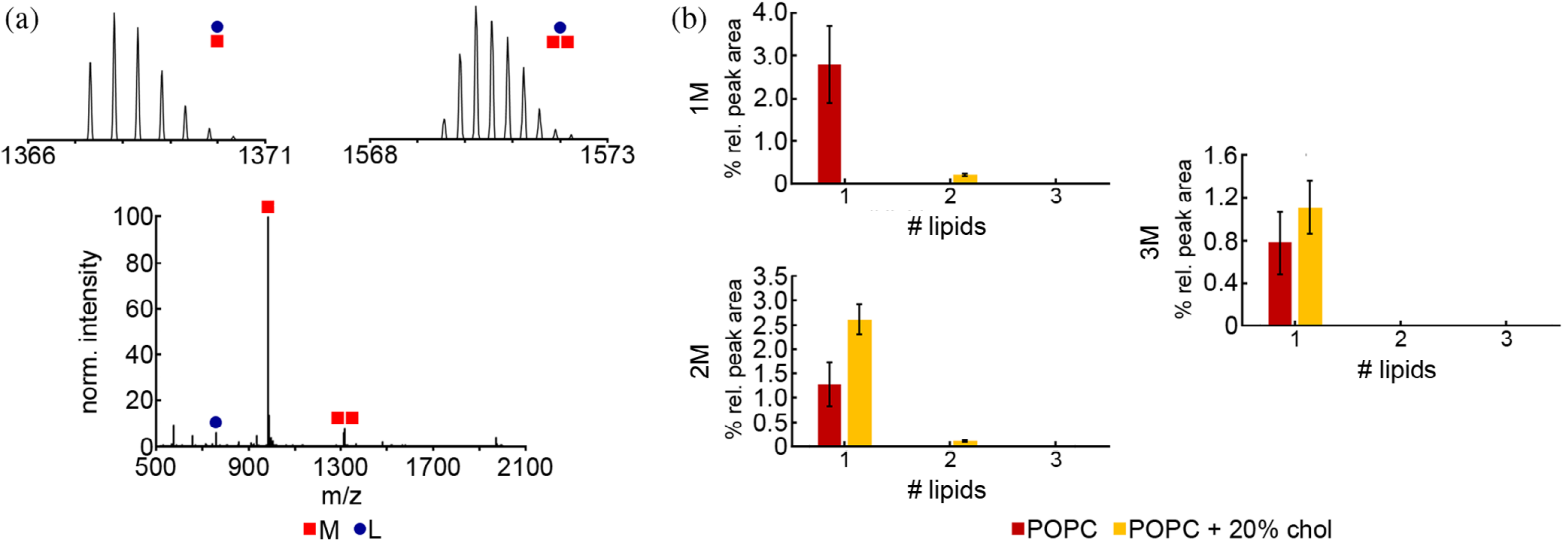
Mass spectrum and analysis of Nt17/lipid complex formation in the presence of pure POPC vesicles and POPC vesicles with 20% exogenously added cholesterol using cVSSI‐MS. Peptides are denoted as M and lipids are denoted as L. The Nt17 concentration was 10 μM and the peptide:lipid ratio was 1:10. (a) A representative mass spectrum of Nt17 incubated with pure POPC vesicles where insets show isotopic distributions of some identified peptide/lipid complexes. (b) The total percent relative peak areas for 1M, 2M, 3M, and 4M complexes identified after incubation with different POPC vesicle compositions. Error bars represent SEM.

When Nt17 was incubated with pure POPC, complexes identified contained upwards of three peptides and one lipid molecule (Table 2; Figure 5a). The highest relative peak area was for [1M + 1L] (2.8% ± 0.90%) and smaller populations of [2M + 1L] and [3M + 1L] (1.3% ± 0.45% and 0.8% ± 0.30%, respectively). When 20% exogenous cholesterol was added to the lipid vesicles, complexes containing upwards of [3M + 1L] and [2M + 2L] were observed (1.1% ± 0.25% and 0.11% ± 0.03%, respectively). Complexes containing one peptide were less prevalent, with a total relative integrated peak area of 0.2%. Complexes containing two and three peptides, however, were more prevalent in the 20% cholesterol system with total relative integrated peaks areas of 2.7% and 1.1%, respectively (Figure 5b).

**TABLE 2:** Identified ions for Nt17 incubated with pure POPC, DOPC, and POPG vesicles and the same vesicles with 20% exogenously added cholesterol with their corresponding mass‐to‐charge ratios and charge states.

Ion^a^	*m*/*z*	Charge state	Ion^a^	*m*/*z*	Charge state	Ion^a^	*m*/*z*	Charge state
*POPC 0% cholesterol*			*DOPC 0% cholesterol*			*POPG 0% cholesterol*		
1Nt17 + 1POPC	1367.31	2	1Nt17 + 1DOPC	1380.32	2	1Nt17 + 1POPG	1361.78	2
2Nt17 + 1POPC	1569.55	3	1Nt17 + 1DOPC	2759.63	1	1Nt17 + 2POPG	1736.05	2
3Nt17 + 1POPC	1670.67	4	1Nt17 + 2DOPC	1773.11	2			
			1Nt17 + 3DOPC	2165.91	2			
			2Nt17 + 1DOPC	1578.22	3			
			2Nt17 + 1DOPC	2366.83	2			
			2Nt17 + 2DOPC	1840.09	3			
			2Nt17 + 3DOPC	2101.95	3			
			3Nt17 + 1DOPC	1677.18	4			
			3Nt17 + 3DOPC	2069.97	4			
			4Nt17 + 1DOPC	2170.43	4			
*POPC + 20% Cholesterol*			*DOPC + 20% Cholesterol*			*POPG + 20% Cholesterol*		
1Nt17 + 2POPC	1747.03	2	1Nt17 + 1DOPC	1380.32	2	1Nt17 + 1POPG	908.19	3
2Nt17 + 1POPC	1569.55	3	1Nt17 + 1DOPC	2759.63	1	1Nt17 + 1POPG	1361.78	2
2Nt17 + 2POPC	1822.74	3	1Nt17 + 2DOPC	1773.11	2	1Nt17 + 3POPG	2110.31	2
3Nt17 + 1POPC	1670.67	4	1Nt17 + 3DOPC	2165.91	2	2Nt17 + 2POPG	1361.78	4
			2Nt17 + 1DOPC	1578.22	3	2Nt17 + 2POPG	1815.38	3
			2Nt17 + 1DOPC	2366.83	2	2Nt17 + 3POPG	1548.91	4
			2Nt17 + 2DOPC	1840.09	3	2Nt17 + 3POPG	2064.88	3
			2Nt17 + 3DOPC	2101.95	3			
			3Nt17 + 1DOPC	1677.18	4			
			3Nt17 + 1DOPC	2235.90	3			
			4Nt17 + 1DOPC	2170.43	4			

^a^ The following monoisotopic masses were used to assign peaks: 1973.03 for Nt17 peptide, 759.58 for POPC lipid, 785.59 for DOPC lipid, and 748.53 for POPG lipid.

**FIGURE 6:**
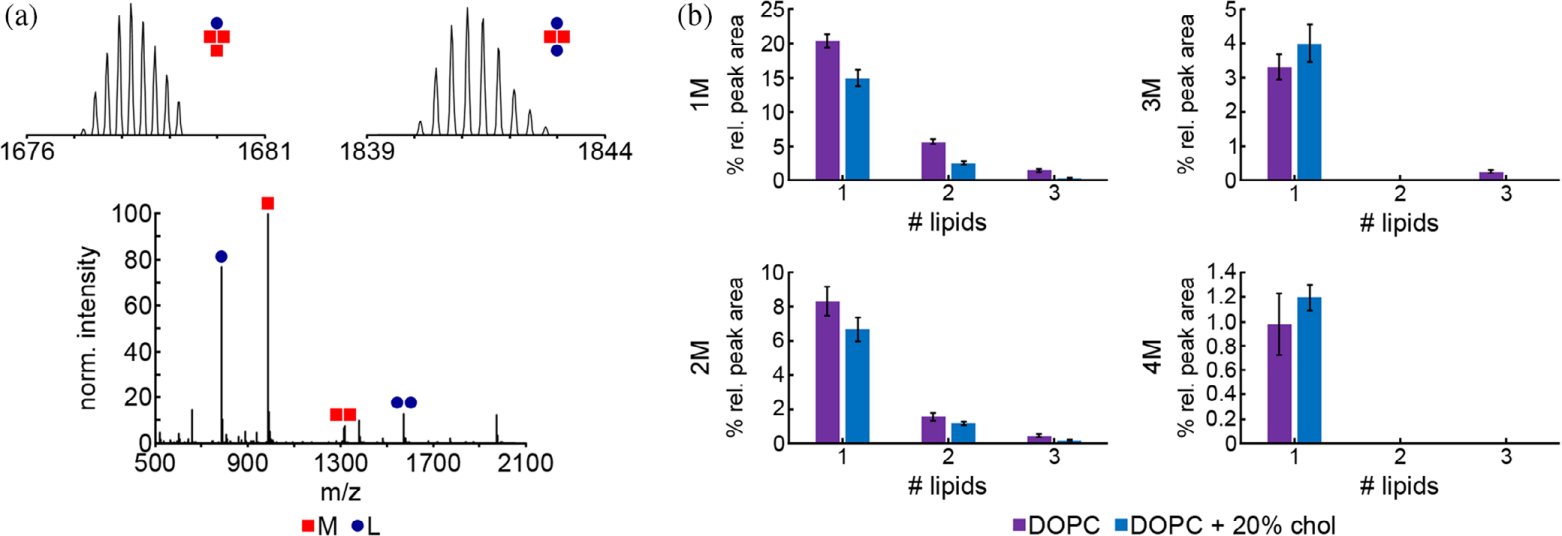
Mass spectrum and analysis of Nt17/lipid complex formation in the presence of pure DOPC vesicles and DOPC vesicles with 20% exogenously added cholesterol using cVSSI‐MS. Peptides are denoted as M and lipids are denoted as L. The Nt17 concentration was 10 μM and the peptide:lipid ratio was 1:10. (a) A representative mass spectrum of Nt17 incubated with pure DOPC vesicles where insets show isotopic distributions of some identified peptide/lipid complexes. (b) The total percent relative peak areas for 1M, 2M, 3M, and 4M complexes identified after incubation with different DOPC vesicle compositions. Error bars represent SEM.

Incubating Nt17 with pure DOPC yielded complexes containing upwards of [4M + 3L] (Table 2; Figure 6a). Complexes containing one peptide account for the largest relative peak area at 27.5% total, followed by those containing two (10.3%), three (3.6%), and four peptides (1.0%). Complexes of up to [1M + 3L] and [2M + 3L] were observed (1.4% ± 0.25% and 0.45% ± 0.07%, respectively) in addition to [3M + 1L] and [4M + 1L] complexes (3.3% ± 0.36% and 0.98% ± 0.25%, respectively). With the addition of 20% exogenous cholesterol, again the largest total relative peak area was associated with complexes containing one peptide (17.7% total relative peak area) followed by two (8.0%), three (4.0%), and four peptides (1.2%). Similar in trend with the pure lipid system, complexes of up to [1M + 3L] and [2M + 3L] were observed (0.27% ± 0.03% and 0.15% ± 0.02%), with decreasing percent relative peak area linked to increasing lipid monomers present in the complex. The complexes containing one or two peptides in the 20% cholesterol system, regardless of the number of lipids attached, were of a lower percent relative peak area compared to the pure lipid system. However, complexes containing three and four peptides, again regardless of the number of lipids in the complex, were of a higher percent relative peak area than those of the pure lipid system (Figure 6b).

**FIGURE 7:**
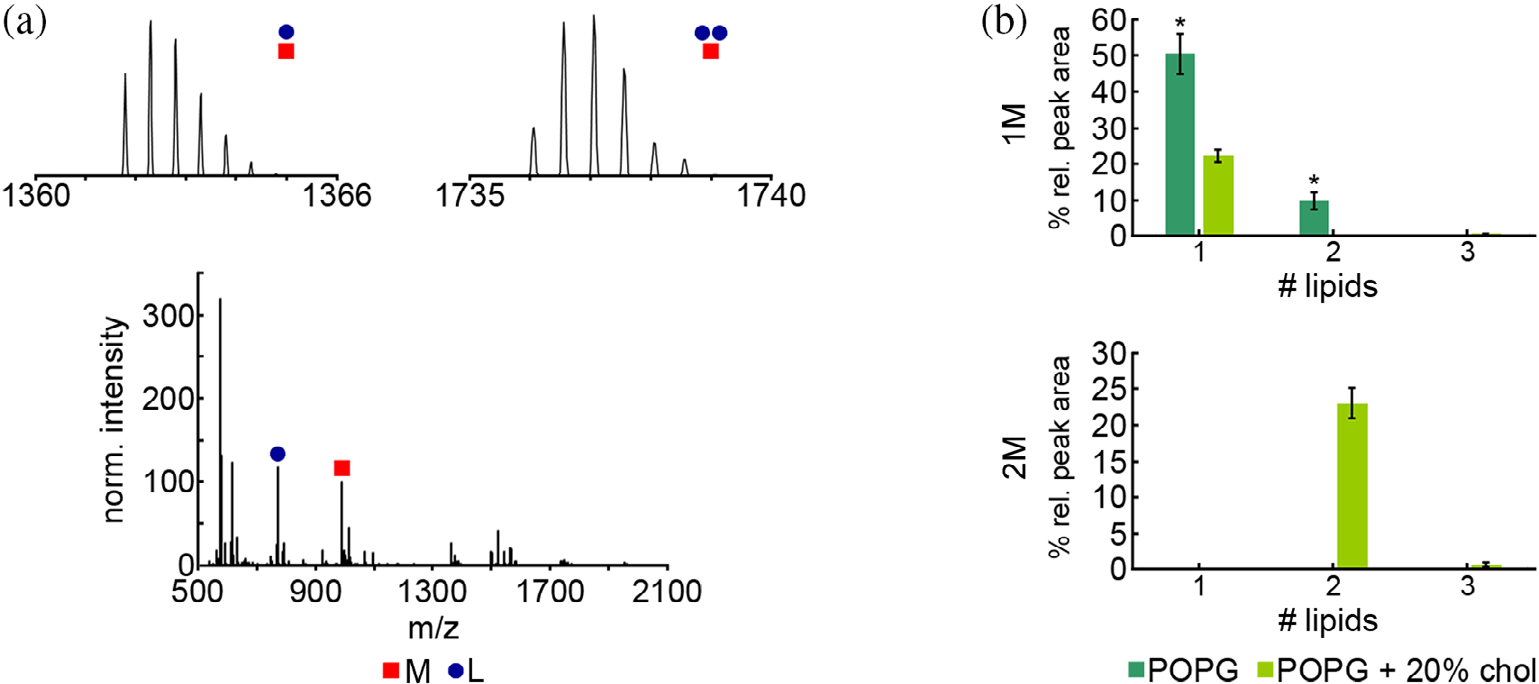
Mass spectrum and analysis of Nt17/lipid complex formation in the presence of pure POPG vesicles and POPG vesicles with 20% exogenously added cholesterol using cVSSI‐MS. Peptides are denoted as M and lipids are denoted as L. The Nt17 concentration was 10 μM and the peptide:lipid ratio was 1:10. (a) A representative mass spectrum of Nt17 incubated with pure POPG vesicles where insets show isotopic distributions of some identified peptide/lipid complexes. (b) The total percent relative peak areas for 1M, 2M, 3M, and 4M complexes identified after incubation with different POPG vesicle compositions. Error bars represent SEM. The * represents conditions where *N* = 2, and the error is represented as the difference between the high and the low values, as the third spectrum in the series shifted toward smaller species indicative of a discharge.

When Nt17 was incubated with pure POPG, peptide/lipid complexes identified contained upwards of [1M + 2L] (Table 2; Figure 7a). The complexes were predominantly [1M + 1L] at 50.5% ± 5.6%, with [1M + 2L] complexes being less prevalent at 9.7% ± 2.5%. When 20% cholesterol was added to the lipid vesicles, complexes containing two peptides were present in addition to complexes containing one peptide. In the 20% cholesterol condition [1M + 1L] complexes were of a lower percent relative peak area at 22.3% ± 1.6%. [2M + 2L] and [2M + 3L] complexes were observed with relative peak areas of 23.0% ± 2.1% and 0.6% ± 0.30%, respectively (Figure 7b).

## 3 DISCUSSION

Amyloid formation and subsequent interactions are influenced by the cellular environment (Aisenbrey et al., 2008; Burke, Yates, & Legleiter, 2013; Gorbenko & Kinnunen, 2006). An important factor is the presence of cellular and subcellular membranes, where membrane composition further modulates amyloid formation and subsequent interactions. In this study, the impacts of cholesterol in simple lipid systems on htt‐exon1(46Q) aggregation and binding were evaluated. In the case of POPC, increasing cholesterol content decreased fibril formation and htt/membrane interactions while promoting dimeric and trimeric Nt17/lipid complexes typically containing a single lipid. Increasing cholesterol content of DOPC vesicles enhanced fibril formation and the ability of htt to bind to the membrane while promoting trimeric and tetrameric htt/lipid complexes that contained fewer lipid monomers. For pure POPG systems, increasing cholesterol content reduced fibril formation, increased htt/lipid interactions, and promoted the formation of dimeric htt/lipid complexes that included more lipid monomers. The data suggests inherently complex htt/lipid interactions and underlies the critical role of lipid composition on aggregation and lipid binding.

Previous studies in conjunction with this study highlight the complexity of htt aggregation mechanisms and lipid interactions (Beasley et al., 2019, 2021, 2022; Chaibva et al., 2018; Côté et al., 2015; Ho et al., 2016; Levy et al., 2019; Pandey et al., 2018). Physiochemical properties of lipids as a function of composition play a dominant role in such mechanisms, and small changes in features such as charge, tail group, or incorporation of other crucial membrane components can have varied impacts. Cholesterol dysregulation has been observed in HD, though debate remains with respect to cholesterol being downregulated (Korade & Kenworthy, 2008; Valenza, 2005; Valenza & Cattaneo, 2011; Vance, 2012) or upregulated (Trushina et al., 2006). Cholesterol is a crucial component of membranes, with important roles in modulating properties such as fluidity and permeability (Subczynski et al., 2017; Wennberg et al., 2012), organization (Arora et al., 2004), and overall membrane function (Arora et al., 2004; Subczynski et al., 2017). Generally, cholesterol preferentially associates with large headgroup lipids, such as PC lipids, and lipids with more saturated tail groups (Huang, 2009). When cholesterol is incorporated into membranes, there are changes to area per lipid, membrane thickness, bending and compressibility moduli, and lipid tail order parameters (Hofsäß et al., 2003; Saeedimasine et al., 2019). In the case of POPC, the addition of cholesterol reduces overall fluidity and increases order (Schumann‐Gillett & O'Mara, 2019), which results in a decrease in membrane defects. This reduction in the presence of defects correlates to reduced lipid binding with increasing cholesterol content observed in the PDA assay. Despite overall reduced lipid binding, with the addition of exogenous cholesterol there was a significant decrease in t_50_ values. This indicates faster interaction between htt and cholesterol‐containing POPC vesicles despite reduced interactions overall. This could suggest that the incorporation of cholesterol influences early interactions of htt with the membrane by facilitating faster association, but due to reduced defects and fluidity overall binding is reduced. Increasing cholesterol content in DOPC lipid systems results in an increase in area per lipid (Alwarawrah et al., 2010), causing more defects at the surface. Increasing the presence of defects correlates to increased lipid binding with increasing cholesterol content observed in the PDA assay. With increasing cholesterol content, there was a significant reduction in t_50_ values relative to the pure lipid system. This suggests that with the addition of cholesterol causing an increase in defects htt more readily associates. POPG lipid systems form a tightly packed bilayer due to interlipid bridges and hydrogen bonding overcoming the electrostatic repulsion that exists between headgroups (Zhao et al., 2007). The addition of cholesterol to POPG systems could spread out the headgroups and allow for more interaction with htt as observed in the PDA assay. The t_50_ values for the POPG systems did not change significantly with the addition of cholesterol, indicating no significant changes in association based on cholesterol content, though overall lipid binding decreases with increasing cholesterol content. This could suggest that initial interactions are driven by electrostatics, but the addition of cholesterol likely spreads out the negative charge over the vesicle surface, reducing htt binding overall. Cholesterol content also varies throughout cellular and subcellular membranes. The plasma membrane contains the largest amount of cholesterol relative to phospholipid content, followed by late endosomes, the golgi, endoplasmic reticulum/nuclear envelope, and the mitochondria (van Meer et al., 2008). The role of cholesterol in modulating physiochemical properties of membranes, its presence and varied content in various cellular and subcellular membrane systems, and its dysregulated homeostasis in the case of HD make it a component of particular interest as its varying impact on membranes can broadly influence the ability of htt to interact with and aggregate on membranes. For example, the addition of cholesterol to brain lipid extract completely alters the morphological impact on bilayers upon exposure to htt‐exon1 (Gao et al., 2016).

The addition of cholesterol altered htt aggregation and the ability to form complexes with all three model systems investigated here. Based on ThT assays, pure POPC had minimal impact on htt‐exon1(46Q) fibril formation, pure DOPC reduced fibril formation, and pure POPG increased fibril formation; trends which align with previous studies (Beasley et al., 2021, 2022). With increasing cholesterol content in the POPC and POPG systems, there was a decrease in fibril formation, while MS revealed Nt17/lipid complexes generally had a higher lipid content. In the case of DOPC, when cholesterol content was increased there was a reduction in fibrilization and MS revealed Nt17/lipid complexes that generally included fewer lipids. These trends suggest that the incorporation of more lipids into Nt17 complexes has a protective role against fibril formation. In the case of htt–lipid interactions revealed by PDA assays, increasing cholesterol content resulted in decreased lipid interactions with POPC systems and increased lipid interactions with DOPC and POPG systems. Trends in lipid binding may be attributed to changes noted in aggregation in the case of POPC and DOPC. With increasing cholesterol content POPC systems showed a reduction in both aggregation and lipid binding, while both trends increased in the case of DOPC. This suggests that with reduced aggregation in the case of POPC systems, interactions with the lipid may be smaller species as indicated by MS data (e.g., the absence of larger tetrameric species). Conversely, larger species including tetramers were present in the case of DOPC systems. The same trend was not observed in the case of POPG, where aggregation decreased with increasing cholesterol content while lipid binding increased. However, it is important to consider the influence cholesterol would have on the number and size of surface defects and, in the case of anionic POPG, charge distribution over the surface of vesicles. It is likely that the inclusion of cholesterol in the systems used for this study also influences Nt17 orientation and insertion.

Based on computational studies, the interaction of Nt17 with phospholipid bilayers involves four basic steps: approach, reorganization, anchoring, and insertion (Côté et al., 2014); however, the behaviors of amyloid proteins and their selective interactions with membranes are heavily dependent on physiochemical properties of the membrane system itself (Burke, Yates, & Legleiter, 2013). In the case of htt specifically, changes to lipid headgroups or tail groups change aggregation, lipid binding, Nt17 orientation at the membrane surface, and htt/lipid complex formation. With anionic lipid headgroups, electrostatics are likely the major influence on htt–lipid interactions, considering the net positive charge of the Nt17 domain (Tao et al., 2019). This is consistent with previous studies demonstrating the localization of htt to acidic membranes (Kegel et al., 2005) and anionic membranes enhancing fibril formation (Beasley et al., 2021). POPC/POPS membranes, for example, enhances htt fibrillization via a unique Nt17‐mediated mechanism based on membrane anchoring and two‐dimensional diffusion (Pandey et al., 2018). Indeed, it appears that anionic character in lipid bilayers can result in a high local concentration at the interface without deep insertion (Beasley et al., 2021), allowing for easy diffusion of htt molecules along the bilayer. This is consistent with the observation of predominately monomeric Nt17 with pure POPG. The altered morphology of both htt oligomers and fibrils observed in the presence of POPG‐based vesicles compared to control also suggests a unique aggregation mechanism. Importantly, this is further mediated by the addition of cholesterol. With POPG, both the PDA assay and the increase in the number of complexes formed suggest that the addition of cholesterol facilitates a deeper penetration of htt into the bilayer. This more intimate interaction with lipids slows htt fibril formation relative to aggregation observed with pure POPG vesicles. Interestingly, the morphological changes of htt aggregates is less pronounced with increasing cholesterol. The heavy influence of anionic lipids on aggregation is observed with other amyloid proteins. For example, acidic phospholipid membranes enhance α‐syn fibrillization (Iyer & Claessens, 2019).

While anionic headgroups promote fibril formation, zwitterionic headgroups have varied impacts on htt fibrilization and indicate stronger htt–lipid interactions, resulting in a higher abundance of complexes containing multiple peptides. With zwitterionic lipids, aggregation was promoted by saturated tail groups, minimally affected by monounsaturated tail groups, and inhibited by polyunsaturated tail groups (Beasley et al., 2022). Computational studies with the same lipid systems demonstrated that the orientation and interactions between Nt17 and lipids also changed as a function of tail saturation, with Nt17 binding more tightly in a parallel orientation in the case of polyunsaturated lipids and less tightly in an orthogonal orientation in the case of saturated lipids (Beasley et al., 2022). In addition to orientation, defect sensing is also a common mechanism associated with amphipathic α‐helix binding to membranes (Drin et al., 2007; Drin & Antonny, 2010; Hatzakis et al., 2009), and this does appear to influence the affinity of Nt17 for membranes comprised of zwitterionic lipids (Beasley et al., 2022). The modifying impact of cholesterol on htt binding both POPC and DOPC vesicles is likely influenced by cholesterol impacting membrane fluidity and packing.

Minor changes to physiochemical properties as a function of lipid composition alters htt aggregation and lipid interactions in unique ways. This is a potential modifying factor to consider with respect to HD as changes to phospholipid metabolism (i.e., PC lipid metabolism) are observed in both HD patients (Mastrokolias et al., 2016; McGarry et al., 2020) and transgenic mouse models (Hashimoto et al., 2021; Tsang et al., 2006) as a function of disease progression. While differentiating individual lipids within a class, that is, saturated versus unsaturated PC lipids, is generally difficult there is interest in pursuing highly unsaturated fatty acids (HUFAs) as a treatment option for HD. The theory behind the approach focuses on potential neuroprotective effects of HUFAs due to their role in membrane function and ability to alter the propensity of cells to undergo apoptosis (Rosser & Dunnett, 2002). Two studies utilizing different HUFAs as treatments for HD patients claimed significant efficacy; however, they are not considered sufficient to guide decisions with respect to treatment due to the small number of participants in each study (Rosser & Dunnett, 2002). Considering the complex nature of physiologically relevant systems whose lipid compositions are unique and likely variable with disease progression, it is important to understand the influence of systems beyond that of pure lipid systems alone to better understand mechanistic interactions of htt with various membranous systems such as organelles and the plasma membrane. The major phospholipid component of most cellular and subcellular membranes are PC lipids, but there are also varying ratios of PE, PI, and PS lipids in addition to others that are difficult to isolate and identify (Subczynski et al., 2017; van Meer et al., 2008; Wennberg et al., 2012). Beyond phospholipid components, there are also varied amounts of other components such as sphingomyelin, gangliosides, and sterols such as cholesterol. In short, the ability of htt to interact with membranes is dependent on numerous factors related to membrane properties, that is, fluidity, packing, thickness, and charge. The relative importance of each factor appears to be highly sensitive to lipid composition.

The ability of htt and its aggregate forms to directly bind and disrupt membranes underscores the development of organelle membrane damage observed in HD. Cytoplasmic htt inclusions formed in mammalian cell and primary neuron models of HD are enriched with and sequester organelle membrane fragments deriving from the ER and mitochondria (Riguet et al., 2021), suggesting that the aggregation process directly damages these surfaces. Indeed, htt fibrils directly impinge upon the ER membrane, compromising its integrity and dynamics (Bäuerlein et al., 2017). In both HD patients and mouse models, htt aggregation exasperates age‐dependent disruption of the nuclear envelope, leading to DNA damage (Gasset‐Rosa et al., 2017). Similar damage to organelle membranes (swollen mitochondria and absent nuclear membrane) develop in the striatum of a novel pig model of HD (Yan et al., 2018). Importantly, each of these organelle membranes have unique lipid compositions. As lipid composition influences how htt interacts with and aggregates on these surfaces, mechanistic details of how htt damages different organelle membranes may vary. Even within mitochondria, htt aggregates impact inner and outer mitochondrial mimic membranes in distinct ways (Adegbuyiro et al., 2021). Understanding how amyloid proteins aggregate in the presence of membranes and interact with them is critical to understanding the intricacies of toxic mechanisms attributable to htt aggregation. The intricacies of these interactions are demonstrated here with simple lipid systems enriched with cholesterol (Table 3). With increasing complexity of membrane composition, these htt/membrane interactions likely will become even more complicated. Future studies with more complex lipid systems could provide further detail on the intricacies of htt interactions with cholesterol‐containing membranes and the relationship between such interactions and toxicity.

**TABLE 3:** Summary of the trends observed with each lipid system for all experiments performed.

Experiment	Results by lipid system		
	POPC	DOPC	POPG
ThT Aggregation Assay	Pure POPC had minimal impact on fibril formation; subsequent additions of cholesterol decreased fibril formation	Pure DOPC reduced fibril formation; subsequent additions of cholesterol increased fibril formation	Pure POPG promoted fibril formation; subsequent additions of cholesterol reduced fibril formation
AFM: Oligomer Morphologies	Pure POPC did not change oligomer morphology; addition of exogenous cholesterol caused significant shift to smaller heights	No significant change to oligomer morphology with or without exogenous cholesterol	Significant shift to smaller oligomer heights both with and without exogenous cholesterol
AFM: Fibril Morphologies	Minimal change to fibril height in the presence of POPC with or without cholesterol	Minimal change to fibril height in the presence of DOPC with or without cholesterol	Shift to slightly smaller fibril heights in the presence of POPG both with and without cholesterol
PDA Lipid Binding Assay	Lipid binding decreased with increasing cholesterol content	Lipid binding increased with increasing cholesterol content	Lipid binding increased with increasing cholesterol content
MS: Peptide/Lipid Complexation	Increased cholesterol content favored dimer and trimer complexes; generally fewer lipids incorporated into complexes	Increased cholesterol content favored trimer and tetramer complexes; generally fewer lipids incorporated into complexes	Increasing cholesterol favored dimer complexes; generally more lipids incorporated into complexes

## 4 MATERIALS AND METHODS

### 4.1 Purification of GST–htt–exon1 fusion protein

Glutathione S‐transferase (GST)–htt–exon1 fusion proteins of disease length (46Q) were purified as previously described (Muchowski et al., 2000). In short, GST‐htt fusion proteins were expressed by induction in *Escherichia coli* with isopropyl‐thio‐galactopyranoside (IPTG) for 4 h at 30°C. The cells were lysed using lysozyme (0.5 mg/mL) and sonication with a sonic dismembrator (FisherSci), then liquid chromatography (BioRad LPLC) using a 5 mL GST affinity column was used to purify the fusion proteins. Sodium dodecyl‐sulfate polyacrylamide gel electrophoresis (SDS‐PAGE) was used to analyze fractions and verify the presence of htt, then the concentration was determined using Coomassie Bradford reagent. Prior to all experiments, high‐speed centrifugation of fusion protein solutions at 20,000*g* at 4°C for 45 min removed pre‐existing aggregates then fusion proteins were incubated with Factor Xa (Promega, Madison, WI) to cleave the GST tag and initiate aggregation for experiments.

### 4.2 Lipid vesicle preparation

Stocks of DOPC, POPC, POPG, and cholesterol were dissolved in chloroform and mixed to the desired mass ratios at a total mass of 1 mg lipid per tube. Once mixed, the chloroform was evaporated off using a gentle stream of nitrogen to form a dried lipid film. Films were stored at −20°C in parafilm‐wrapped Eppendorf tubes until needed. On the day of experimentation, lipid films were rehydrated in tris buffer (pH = 7.4) with agitation for a minimum of 1 h. Lipid vesicles were formed via 10 freeze–thaw cycles using liquid nitrogen and a thermomixer (45°C), followed by 1 h of bath sonication.

### 4.3 Thioflavin T aggregation assay

Thioflavin T (ThT) assays were used to monitor htt fibril formation as a function of time in the presence and absence of different lipid systems. For each condition, htt‐exon1(46Q) (10 μM) was incubated with ThT (125 μM) in black Costar 96‐well plates with clear flat bottoms. For the lipid conditions, 100 μM of the desired lipid vesicle solution was added to result in a 10:1 lipid:protein ratio. Experiments were run at 37°C for 18 h, and ThT fluorescence was recorded every 5 min using a SpectraMax M2 microplate reader (excitation 400 nm, emission 484 nm). Relative maximum fluorescence was calculated by normalizing the maximum fluorescence intensity of each condition to the maximum fluorescence of the huntingtin control (100%). Relevant controls were measured and subsequently subtracted from the curves to show the fluorescent signal strictly associated with htt aggregation. Each condition was performed a minimum of three times, and error bars represent the standard error.

### 4.4 Polydiacetylene lipid binding assay

To measure htt–lipid interaction, polydiacetylene (PDA) lipid binding assays were performed using previously reported protocols (Sokolovski et al., 2008; Zheng et al., 2012). Briefly, monomeric 10,12‐tricosadiynoic acid was mixed with the desired lipid system at a 2:3 molar ratio in a 4:1 chloroform/ethanol solution. Lipid films used in this step were formed by mixing lipids to the desired cholesterol content and then drying, as previously described. Once the lipid and 10,12‐tricosadiynoic acid were mixed, the organic solvents were evaporated off with a gentle stream of nitrogen. Resulting films were rehydrated in tris buffer (70°C) and sonicated to promote mixing. Periods of sonication did not exceed 5 min each to avoid extensive heating of the solution. Following sonication, lipid solutions were left at 4°C overnight to allow for PDA/lipid vesicle formation. The following day, lipid solutions were equilibrated to 25°C and irradiated at 254 nm to polymerize the 10,12‐tricosadiynoic acid, resulting in a royal blue solution that would undergo a colorimetric shift to red when stress was applied to the vesicles. PDA/lipid vesicles were then incubated with htt–exon1(46Q) (10 μM) for 18 h at 30°C. Absorbance of the blue (650 nm) and red (500 nm) wavelengths were recorded every 5 min on a SpectraMax M2 microplate reader with 1 min of orbital shaking before each read. Negative controls consisted of equal parts PDA/lipid solution and neat buffer for each unique lipid mixture. As a positive control, PDA/lipid vesicles were incubated with equal parts saturated NaOH (pH = 12), which induces a colorimetric response by increasing repulsion among lipid head groups and causing stress on the vesicles (Jelinek & Kolusheva, 2001, 2007). The NaOH results in the maximum colorimetric shift for each lipid system that can then be used to establish the sensitivity of different lipid/PDA systems and normalize results (Beasley et al., 2020). For each condition, the percent colorimetric response (% CR) was calculated using the following equation:

$$\%\mathrm{CR}=\left(\frac{{\mathrm{PB}}_{0}-\mathrm{PB}}{{\mathrm{PB}}_{0}}\right)\times 100$$

where PB is defined as *A*_blue_/(*A*_blue_ + *A*_red_) for the negative control (PB_0_) and sample condition (PB). Each condition was performed a minimum of three times, and error bars represent the standard error of the sample set.

### 4.5 Atomic force microscopy

Purified htt–exon1(46Q) (10 μM) was incubated with and without lipid vesicles (100 μM for a 10:1 lipid:protein ratio) at 37°C with constant orbital agitation. At the desired time points, 2 μL of each sample was deposited on freshly cleaved mica. One minute after deposition, the mica was rinsed with 200 μL of 18 MΩ water and dried with a gentle stream of clean air. Samples were imaged using a Nanoscope V Multi‐Mode scanning probe microscope (VEECO) equipped with a closed loop vertical engage J‐scanner. Silicon‐cantilevers with a nominal spring constant of 40 N/m and a resonance frequency of 300 kHz were used. All images were collected with a scan rate of 1.99 Hz and cantilever drive frequencies at 10%–20% of resonance. Images were analyzed using the Matlab image processing toolbox (MathWorks) as previously described (Burke & Legleiter, 2013).

### 4.6 Pulled‐tip capillary emitter and cVSSI device fabrication

Detailed descriptions of cVSSI devices have been provided previously (Li et al., 2020; Ranganathan et al., 2019). In short, a piezoelectric transducer was attached to the end of a glass slide coverslip using epoxy glue. A glass capillary emitter (0.5 mm I.D.) was pulled (Sutter Instrument Company, Novato, CA) and mechanically cut with a ceramic cutter to the desired I.D. (70–100 μm) verified under a microscope. The emitter was secured to the end opposite the piezoelectric transducer using glass glue. A blunt, fused silica capillary (250 μm I.D., 355 μm O.D., 5 cm‐long) was inserted into the glass emitter and glued to the flat end using epoxy glue. PTFE tubing was used to connect a syringe to the fused silica capillary of the cVSSI device. A 5‐cm platinum wire was inserted into the PTFE tubing near the end connecting to the cVSSI device as an electrode for field‐enabled experiments. RF signal (~94 kHz sine wave, 9–10 V_pp_) was applied to the piezoelectric transducer via a function generator and connected amplifier.

### 4.7 cVSSI‐mass spectrometry

MS was used to investigate complexes formed between Nt17 peptide and various lipid vesicles with the different cholesterol content. Lipid vesicles were formed by rehydrating films in 10 mM ammonium acetate solution, bath sonicated for 1 h, and subjected to 10 freeze–thaw cycles using liquid nitrogen. Nt17 peptide (10 μM) was incubated with lipid vesicles (10:1 lipid:peptide ratio) for 24 h at 37°C. Samples were analyzed using a Q‐Exactive Hybrid Quadrupole mass spectrometer using cVSSI devices. Spectra were collected in both positive‐ion and negative‐ion modes over a mass‐to‐charge (*m*/*z*) ratio range of 350–4000. Samples were infused at a flow rate of 10 μL/min with 1.8 kV applied to the Pt wire. MS instrument parameters were 250°C capillary inlet temperature, 1 × 10^6^ for the AGC, and 70,000 for MS resolution. Mass spectra for each sample were recorded in triplicate for 30 s each, with the high voltage turned off and the device flushed between each replicate to account for any device variability. Data were analyzed using the Xcalibur 2.2 software suite (Thermo Scientific).

## AUTHOR CONTRIBUTIONS

**Alyssa Stonebraker:** Conceptualization (equal); formal analysis (equal); investigation (equal); validation (equal); visualization (equal); writing – original draft (lead); writing – review and editing (equal). **Maryssa Beasley:** Conceptualization (equal); data curation (equal); formal analysis (equal); investigation (equal); validation (equal); visualization (equal); writing – review and editing (equal). **Sophia Massinople:** Formal analysis (supporting); writing – review and editing (supporting). **Michelle Wunder:** Formal analysis (supporting); writing – review and editing (supporting). **Peng Li:** Funding acquisition (equal); methodology (equal); resources (equal); writing – review and editing (supporting). **Stephen Valentine:** Formal analysis (equal); methodology (equal); supervision (equal); writing – review and editing (equal). **Justin Legleiter:** Conceptualization (equal); data curation (equal); project administration (lead); software (equal); visualization (supporting); writing – original draft (supporting); writing – review and editing (equal).

## Data Availability

The data that support the findings of this study are available from the corresponding author upon reasonable request.
